# Supplementary material for: Diversity in Fruit Morphology and Nutritional Composition of Juglans mandshurica Maxim in Northeast China
Source: Front Plant Sci. 2022 Feb 10;13:820457. doi: 10.3389/fpls.2022.820457 (PMC8866725; doi:10.3389/fpls.2022.820457)
Supplement: Supplementary file 1 [file Data_Sheet_1.docx]

Supplemental Figures and Tables

[Supplemental Figure 1 1](#_Toc87868058)

[Supplemental Figure 2 2](#_Toc87868059)

[Supplemental Figure 3 3](#_Toc87868060)

[Supplemental Table S1 4](#_Toc87868061)

[Supplemental Table S2 4](#_Toc87868062)

[Supplemental Table S3 4](#_Toc87868063)

[Supplemental Table S4 4](#_Toc87868064)

[Supplemental Table S5 4](#_Toc87868065)

[Supplemental Table S6 4](#_Toc87868066)

# Supplemental Figure 1


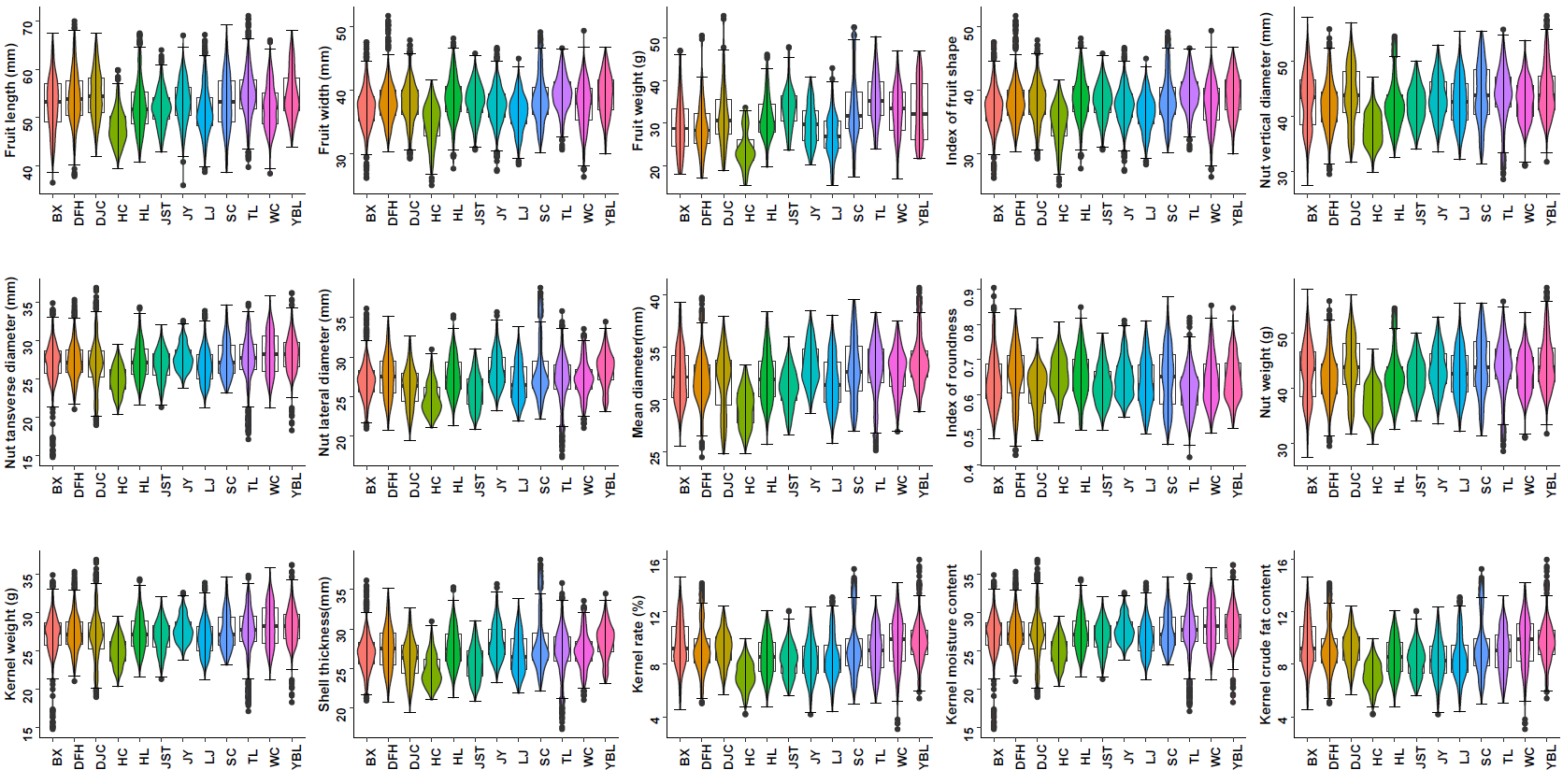


**Figure S1.** Distribution of fruit morphological traits.

# Supplemental Figure 2


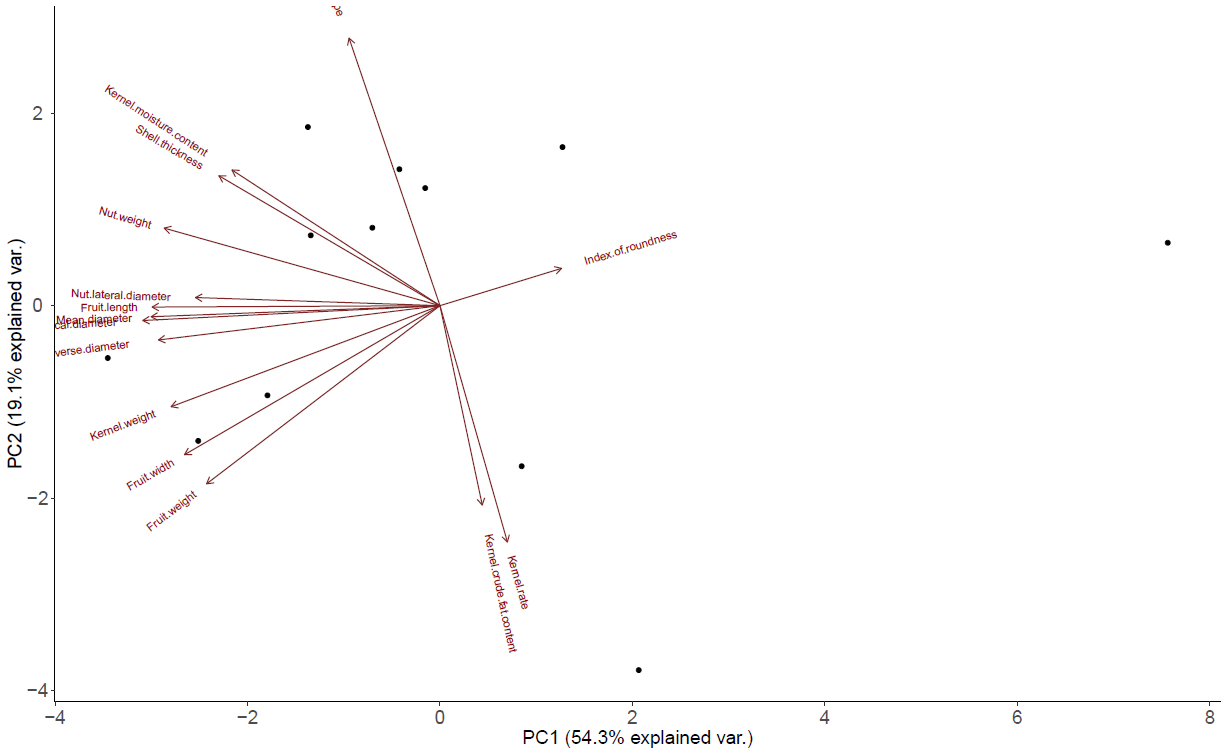


**Figure S2.** Principle component analysis using fruit morphology

# Supplemental Figure 3


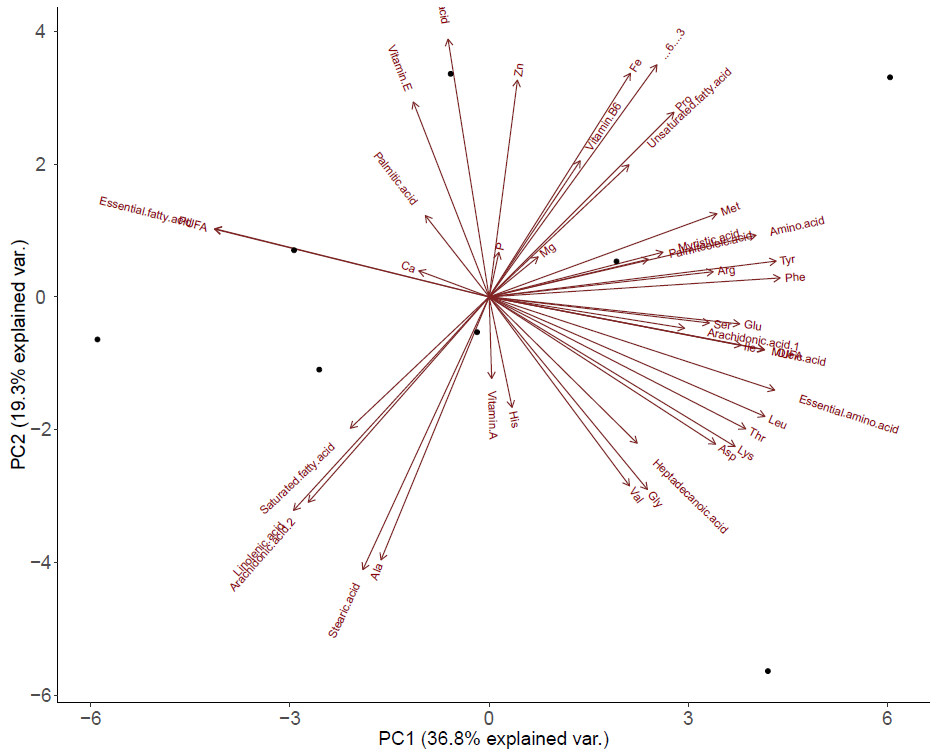


**Figure S3.** Principle component analysis using kernel nutritional composition
