# Supplementary material for: Diversity in Fruit Morphology and Nutritional Composition of Juglans mandshurica Maxim in Northeast China
Source: Front Plant Sci. 2022 Feb 10;13:820457. doi: 10.3389/fpls.2022.820457 (PMC8866725; doi:10.3389/fpls.2022.820457)
Supplement: Supplementary file 2 [file Table_1.DOCX]

**Table S1.** Geographical and ecological conditions for 12 *J. mandshurica* populations

| **Site** | **Code** | **LAT (°N)** | **LON (°E)** | **ALT (m)** | **MAT** | **AT5** | **AT10** | **AP (mm)** | **DL (h)** | **Sample size** |
| --- | --- | --- | --- | --- | --- | --- | --- | --- | --- | --- |
| Binxian, HLJ | BX | 45.34 | 127.35 | 389 | 4.3 | 3124.2 | 2776.6 | 533.6 | 2504.4 | 31 |
| Raohe county, HLJ | DFH | 46.30 | 133.44 | 139 | 3.1 | 3040.9 | 2689.5 | 699.3 | 2492.1 | 28 |
| Ning'an, HLJ | DJC | 43.51 | 129.10 | 522 | 4.2 | 3060.9 | 2738.5 | 411.5 | 2538.2 | 26 |
| Hunchun City, JL | HC | 43.15 | 131.13 | 300 | 7.4 | 3462.8 | 3006.6 | 457.2 | 2434.6 | 20 |
| Hulin City, HLJ | HL | 45.58 | 132.19 | 225 | 4.1 | 3193.7 | 2752 | 647.7 | 2896.1 | 26 |
| Yichun City, HLJ | JST | 47.23 | 129.37 | 302 | 1.8 | 2708.6 | 2350.1 | 730.7 | 2304.1 | 30 |
| Jiayin City, HLJ | JY | 48.28 | 130.35 | 119 | 0 | 2802.9 | 2567.8 | 647.6 | 2489.7 | 25 |
| Linjiang City, JL | LJ | 41.56 | 126.59 | 637 | 5.9 | 3251.3 | 2658.2 | 583.7 | 2146.2 | 30 |
| Baishan City, JL | SC | 42.31 | 126.46 | 583 | 4.1 | 2763.5 | 2463.8 | 701.9 | 2354.3 | 30 |
| Tieli City, HLJ | TL | 47.28 | 128.14 | 362 | 2.2 | 2873.1 | 2637.7 | 950.8 | 2411.3 | 30 |
| Wuchang City, HLJ | WC | 44.54 | 127.49 | 242 | 4.9 | 3357.6 | 2830.1 | 562.1 | 2148.8 | 30 |
| Yabuli, HLJ | YBL | 44.57 | 128.50 | 383 | 4 | 3102.4 | 2761.9 | 544.4 | 2248.6 | 30 |

**ALT**, altitude; **AP**, annual precipitation; **AT5**:accumulated temperature above 5℃; **AT10**:Accumulated temperature above 10℃; **DL**: day length; **HLJ**: Heilongjiang Province; **JL**: Jilin Province; **LAT**, latitude; **LON**, longitude; **MAT**, annual mean air temperature.
