# Supplementary material for: Diversity in Fruit Morphology and Nutritional Composition of Juglans mandshurica Maxim in Northeast China
Source: Front Plant Sci. 2022 Feb 10;13:820457. doi: 10.3389/fpls.2022.820457 (PMC8866725; doi:10.3389/fpls.2022.820457)
Supplement: Supplementary file 4 [file Table_3.DOCX]

**Table S3.** Inter- and intra-group variations of fruit morphology of 12 *J. mandshurica* populations

| **Fruit morphology** | **Variance（df）** | | | **F values** | |
| --- | --- | --- | --- | --- | --- |
|  | **Inter-group** | **Intra-group** | **Random error** | **Inter-group** | **Intra- group** |
| Fruit length | 2476.441（11） | 535.189（324） | 6.346（9317） | 390.218^**^ | 84.331^**^ |
| Fruit width | 1117.114（11） | 210.064（324） | 3.084（9317） | 362.253^**^ | 68.119^**^ |
| Index of fruit shape | 0.537（11） | 0.373（324） | 0.006（9317） | 90.59^**^ | 63.024^**^ |
| Fruit weight | 999.813（11） | 140.297（324） | 2.547（927） | 392.487^**^ | 55.075^**^ |
| Nut vertical diameter | 1816.411（11） | 439.199（321） | 4.067（8648） | 446.597^**^ | 107.985^**^ |
| Nut transverse diameter | 479.785（11） | 141.196（321） | 1.117（8648） | 429.527^**^ | 126.405^**^ |
| Nut lateral diameter | 616.457（11） | 143.601（228） | 1.155（6100） | 533.744^**^ | 124.333^**^ |
| Mean diameter | 706.863（11） | 133.747（228） | 0.989（6100） | 715.08^**^ | 135.302^**^ |
| Index of roundness | 0.11（11） | 0.096（228） | 0.001（6100） | 121.248^**^ | 105.866^**^ |
| Shell thickness | 65.199（11） | 6.338（237） | 0.178（5499） | 367.206^**^ | 35.698^**^ |
| Nut weight | 287.919（11） | 51.049（237） | 0.665（5499） | 432.668^**^ | 76.714^**^ |
| Kernel weight | 8.187（11） | 1.835（237） | 0.035（5499） | 235.13^**^ | 52.717^**^ |
| Kernel rate | 0.055（11） | 0.011（237） | 0.0002（5499） | 283.896^**^ | 55.145^**^ |

*: p < 0.05; **: p < 0.01.
