# Supplementary material for: Diversity in Fruit Morphology and Nutritional Composition of Juglans mandshurica Maxim in Northeast China
Source: Front Plant Sci. 2022 Feb 10;13:820457. doi: 10.3389/fpls.2022.820457 (PMC8866725; doi:10.3389/fpls.2022.820457)
Supplement: Supplementary file 5 [file Table_4.DOCX]

**Table S4.** Coefficient of variation of fruit morphology in 12 *J. mandshurica* populations

| Population | FL | FW | IF | FT | NV | NT | NL | DM | IR | NW | KW | TS | KR | Mean |
| --- | --- | --- | --- | --- | --- | --- | --- | --- | --- | --- | --- | --- | --- | --- |
| BX | 10.58 | 8.71 | 9.86 | 22.92 | 12.08 | 10.15 | 8.91 | 8.06 | 11.52 | 19.16 | 19.18 | 10.53 | 12.79 | 12.65 |
| DFH | 10.09 | 8.70 | 11.20 | 22.03 | 10.20 | 8.49 | 10.04 | 7.54 | 12.29 | 19.60 | 22.96 | 10.35 | 15.65 | 13.01 |
| DJC | 9.16 | 8.35 | 8.53 | 22.63 | 11.99 | 11.95 | 9.56 | 9.28 | 9.37 | 14.06 | 15.74 | 13.09 | 13.49 | 12.09 |
| HC | 7.51 | 9.05 | 10.62 | 17.95 | 8.19 | 7.20 | 7.74 | 6.24 | 7.89 | 14.58 | 16.25 | 8.67 | 8.14 | 10.00 |
| HL | 9.97 | 8.51 | 10.65 | 16.82 | 9.94 | 8.56 | 8.51 | 7.43 | 9.94 | 15.99 | 19.93 | 12.84 | 14.78 | 11.84 |
| JST | 7.05 | 6.99 | 7.56 | 14.50 | 7.83 | 7.37 | 8.33 | 5.80 | 8.57 | 14.06 | 14.47 | 14.30 | 10.62 | 9.80 |
| JY | 8.04 | 7.69 | 8.51 | 15.90 | 8.62 | 5.88 | 8.00 | 5.93 | 8.31 | 15.94 | 22.49 | 10.98 | 16.18 | 10.96 |
| LJ | 9.32 | 7.67 | 9.73 | 19.32 | 10.27 | 8.15 | 9.12 | 7.84 | 10.76 | 18.63 | 18.00 | 11.15 | 11.66 | 11.66 |
| SC | 11.00 | 9.42 | 9.46 | 24.50 | 12.28 | 8.86 | 12.55 | 9.11 | 13.29 | 22.36 | 19.33 | 12.40 | 11.14 | 13.52 |
| TL | 8.96 | 7.08 | 9.99 | 17.03 | 10.83 | 10.99 | 10.82 | 8.08 | 9.94 | 18.20 | 16.25 | 10.23 | 11.44 | 11.53 |
| WC | 8.66 | 9.42 | 10.71 | 18.28 | 8.64 | 9.86 | 8.11 | 6.47 | 10.62 | 17.71 | 22.84 | 9.52 | 20.48 | 12.41 |
| YBL | 9.06 | 8.31 | 8.62 | 21.81 | 10.32 | 8.30 | 7.59 | 6.74 | 9.46 | 16.81 | 15.11 | 11.40 | 12.38 | 11.22 |
| Mean* | 9.79  bc | 8.85  ab | 9.81  bc | 22.00  g | 10.92  cd | 9.51  bc | 10.06  bc | 8.26  a | 10.52  cd | 19.42  f | 19.89  fg | 13.14  d | 14.32  e | 12.81 |

**FL**: Fruit length (mm); **FW**: Fruit width (mm); **IF**: Index of fruit shape; **FT**: Fruit weight (g); **NV**: Nut vertical diameter (mm); **NT**: Nut transverse diameter (mm); **NL**: Nut lateral diameter (mm); **DM**: Mean diameter (mm); **TS**: Shell thickness (mm); **IR**: Index of roundness; **NW**: Nut weight (g); **KW**: Kernel weight (g); **KR**: Kernel rate (g); * mean of the trait; different letters denote statistical significance.
