# Supplementary material for: Diversity in Fruit Morphology and Nutritional Composition of Juglans mandshurica Maxim in Northeast China
Source: Front Plant Sci. 2022 Feb 10;13:820457. doi: 10.3389/fpls.2022.820457 (PMC8866725; doi:10.3389/fpls.2022.820457)
Supplement: Supplementary file 6 [file Table_5.DOCX]

**Table S5.** Percent relative range of fruit morphology in 12 *J. mandshurica* populations

| Population | FL | FW | IF | FT | NV | NT | NL | DM | IR | NW | KW | Mean* | TS | KR | Mean** |
| --- | --- | --- | --- | --- | --- | --- | --- | --- | --- | --- | --- | --- | --- | --- | --- |
| BX | 87.98 | 80.11 | 84.97 | 72.81 | 99.06 | 90.93 | 70.87 | 84.80 | 89.20 | 78.58 | 80.27 | 83.60^d^ | 44.89 | 67.25 | 56.07 |
| DFH | 91.53 | 80.08 | 85.69 | 84.29 | 85.60 | 64.62 | 67.32 | 94.18 | 86.36 | 78.01 | 90.92 | 82.60^d^ | 45.47 | 72.87 | 59.17 |
| DJC | 72.65 | 69.87 | 73.17 | 91.46 | 82.58 | 81.09 | 61.58 | 81.36 | 60.72 | 65.83 | 50.05 | 71.85^bc^ | 70.11 | 55.26 | 62.69 |
| HC | 57.99 | 61.99 | 76.51 | 45.66 | 56.11 | 41.43 | 46.36 | 52.09 | 59.10 | 49.88 | 43.62 | 53.70^a^ | 28.69 | 43.09 | 35.89 |
| HL | 76.09 | 76.81 | 69.45 | 66.49 | 71.72 | 57.45 | 65.27 | 78.25 | 72.43 | 52.20 | 64.60 | 68.25^bc^ | 42.41 | 65.13 | 53.77 |
| JST | 60.26 | 56.85 | 57.34 | 60.94 | 52.19 | 48.42 | 47.76 | 58.09 | 57.19 | 47.26 | 53.33 | 54.51^a^ | 63.25 | 66.77 | 65.01 |
| JY | 88.40 | 72.76 | 89.32 | 51.94 | 63.31 | 39.67 | 58.31 | 60.86 | 57.31 | 72.15 | 77.23 | 66.48^b^ | 46.88 | 84.21 | 65.54 |
| LJ | 81.01 | 62.51 | 79.90 | 69.16 | 75.84 | 56.99 | 55.60 | 75.87 | 66.54 | 69.47 | 53.30 | 67.84^bc^ | 55.19 | 52.49 | 53.84 |
| SC | 87.07 | 71.14 | 76.32 | 88.25 | 78.32 | 52.08 | 77.59 | 77.65 | 87.34 | 81.92 | 77.29 | 77.72^cd^ | 51.76 | 48.68 | 50.22 |
| TL | 89.26 | 59.89 | 83.71 | 66.37 | 88.36 | 80.05 | 86.23 | 81.59 | 82.35 | 65.83 | 57.10 | 76.43^bcd^ | 41.17 | 58.97 | 50.07 |
| WC | 78.65 | 86.27 | 82.60 | 75.44 | 74.12 | 66.29 | 58.45 | 65.18 | 75.52 | 74.70 | 74.44 | 73.79^bc^ | 39.52 | 79.47 | 59.50 |
| YBL | 68.73 | 63.00 | 65.93 | 63.50 | 86.15 | 81.05 | 52.99 | 73.35 | 70.89 | 89.45 | 60.42 | 70.50^bc^ | 55.97 | 66.07 | 61.02 |

**FL**: Fruit length (mm); **FW**: Fruit width (mm); **IF**: Index of fruit shape; **FT**: Fruit weight (mm); **NV**: Nut vertical diameter (mm); **NT**: Nut transverse diameter (mm); **NL**: Nut lateral diameter (mm); **DM**: Mean diameter (mm); **TS**: Shell thickness (mm); **IR**: Index of roundness; **NW**: Nut weight (g); **KW**: Kernel weight (g); **KR**: Kernel rate (g); * mean of the 11 traits on the left; different letters denote statistical significance; ** mean of the two traits (TS and KR).
