# Supplementary material for: Diversity in Fruit Morphology and Nutritional Composition of Juglans mandshurica Maxim in Northeast China
Source: Front Plant Sci. 2022 Feb 10;13:820457. doi: 10.3389/fpls.2022.820457 (PMC8866725; doi:10.3389/fpls.2022.820457)
Supplement: Supplementary file 7 [file Table_6.DOCX]

**Table S6.** Inter- and intra-group variations of kernel nutritional compositions

| **Nutritional**  **compositions** | **Variance（df）** | | | **F values** | |
| --- | --- | --- | --- | --- | --- |
|  | **Inter-group** | **Intra-group** | **Random error** | **Inter-group** | **Intra- group** |
| Crude fat | 0.04（11） | 0.005（235） | 0.0003（494） | 146.38^**^ | 19.61^**^ |
| H_2_O | 0.004（11） | 0.0003（235） | 2.56E-05（494） | 159.76^**^ | 12.35^**^ |
| Myristic acid | 2.66E-07（7） | 1.10E-07（112） | 2.74E-09（240） | 97.08^**^ | 40.28^**^ |
| Palmitic acid | 3.60E-05（7） | 2.22E-05（112） | 1.58E-07（240） | 227.56^**^ | 139.93^**^ |
| Heptadecanoic acid | 1.64E-08（7） | 2.64E-09（112） | 1.36E-10（240） | 120.50^**^ | 19.35^**^ |
| Stearic acid | 2.40E-05（7） | 4.72E-06（112） | 8.58E-09（240） | 2800.88^**^ | 550.16^**^ |
| Saturated fatty acid | 4.14E-05（7） | 3.23E-05（112） | 1.89E-07（240） | 219.41^**^ | 170.98^**^ |
| Linoleic acid | 0.007（7） | 0.002（112） | 5.53E-06（240） | 1197.37^**^ | 306.30^**^ |
| alpha-linolenic acid | 0.006（7） | 0.001（112） | 4.27E-07（240） | 13823.57^**^ | 2757.44^**^ |
| Essential fatty acids | 0.004（7） | 0.004（112） | 3.35E-06（240） | 1340.87^**^ | 1066.08^**^ |
| Arachidonic acid | 1.97E-08（7） | 1.55E-08（112） | 2.33E-10（240） | 84.34^**^ | 66.36^**^ |
| Polyunsaturated fatty acid | 0.004（7） | 0.004（112） | 3.33E-06（240） | 1346.66^**^ | 1072.80^**^ |
| Palmitonoic acid | 5.66E-08（7） | 4.51E-08（112） | 2.35E-10（240） | 240.26^**^ | 191.57^**^ |
| Oleic acid | 0.005（7） | 0.003（112） | 2.67E-06（240） | 1801.69^**^ | 1305.16^**^ |
| Arachidonic acid | 6.67E-07（7） | 2.88E-07（112） | 1.38E-09（240） | 483.04^**^ | 208.43^**^ |
| Monounsaturated fatty acid | 0.005（7） | 0.004（112） | 2.75E-06（240） | 1789.04^**^ | 1275.96^**^ |
| Unsaturated fatty acid | 4.14E-05（7） | 3.23E-05（112） | 1.90E-07（240） | 217.97^**^ | 169.78^**^ |
| ω-6/ω-3 | 15.31（7） | 2.04（112） | 0.002（240） | 7088.09^**^ | 944.49^**^ |
| Essential amino acid | 6.641（7） | 2.583（112） | 0.055（240） | 121.07^**^ | 47.09^**^ |
| Thr | 0.153（7） | 0.035（112） | 0.002（240） | 80.85^**^ | 18.57^**^ |
| Val | 0.097（7） | 0.101（112） | 0.007（240） | 13.30^**^ | 13.85^**^ |
| Ile | 0.241（7） | 0.101（112） | 0.003（240） | 83.83^**^ | 35.34^**^ |
| Leu | 0.455（7） | 0.145（112） | 0.009（240） | 51.57^**^ | 16.43^**^ |
| Phe | 0.557（7） | 0.193（112） | 0.009（240） | 64.51^**^ | 22.33^**^ |
| Lys | 0.07（7） | 0.035（112） | 0.003（240） | 27.29^**^ | 13.90^**^ |
| Asp | 0.888（7） | 0.259（112） | 0.02（240） | 45.28^**^ | 13.21^**^ |
| Ser | 0.548（7） | 0.093（112） | 0.003（240） | 170.25^**^ | 28.85^**^ |
| Glu | 10.291（7） | 1.584（112） | 0.108（240） | 95.58 ^**^ | 14.71^**^ |
| Gly | 0.322（7） | 0.174（112） | 0.006（240） | 55.71^**^ | 30.08^**^ |
| Ala | 1.369（7） | 0.168（112） | 0.01（240） | 130.99^**^ | 16.10^**^ |
| Met | 0.337（7） | 0.124（112） | 0.002（240） | 212.02^**^ | 77.79^**^ |
| Tyr | 0.241（7） | 0.1（112） | 0.003（240） | 77.96^**^ | 32.50^**^ |
| His | 0.336（7） | 0.082（112） | 0.003（240） | 113.75^**^ | 27.71^**^ |
| Arg | 5.184（7） | 1.169（112） | 0.08（240） | 64.68^**^ | 14.59^**^ |
| Pro | 105.279（7） | 13.475（112） | 0.131（240） | 801.07^**^ | 102.53^**^ |
| Total Amino acid | 294.753（7） | 39.437（112） | 0.963（240） | 306.20^**^ | 40.97^**^ |
| Vitamin_B6 | 90.651（7） | 3.333（112） | 0.095（240） | 952.79^**^ | 35.03^**^ |
| Vitamin E | 5.075（7） | 0.813（112） | 0.001（240） | 5222.90^**^ | 837.24^**^ |
| Vitamin A | 69589（7） | 17440（112） | 28.0（240） | 2488.79^**^ | 623.72^**^ |
| Fe | 2538.3（7） | 181.6（112） | 9.7（240） | 261.95^**^ | 18.74^**^ |
| Zn | 982.3（7） | 85.3（112） | 5.5（240） | 180.22^**^ | 15.65^**^ |
| Ca | 407164（7） | 77396（112） | 3650（240） | 111.56^**^ | 21.21^**^ |
| Mg | 3321195（7） | 292374（112） | 14248（240） | 233.10^**^ | 20.52^**^ |
| P | 21497736（7） | 1381161（112） | 71144（240） | 302.17^**^ | 19.41^**^ |

*: p < 0.05; **: p < 0.01.
