# Supplementary material for: Diversity in Fruit Morphology and Nutritional Composition of Juglans mandshurica Maxim in Northeast China
Source: Front Plant Sci. 2022 Feb 10;13:820457. doi: 10.3389/fpls.2022.820457 (PMC8866725; doi:10.3389/fpls.2022.820457)
Supplement: Supplementary file 8 [file Table_7.DOCX]

**Table S7.** Correlation between geographical/ecological factors on fruit morphology and nutrient compositions

|  | **LON** | **LAT** | **ALT** | **ELAT** | **MAT** | **AT5** | **AT10** | **AP** | **DL** |
| --- | --- | --- | --- | --- | --- | --- | --- | --- | --- |
| Fruit length | -0.019 | 0.120* | 0.005 | 0.188** | -0.197** | -0.172** | -0.092 | 0.108* | 0.037 |
| Fruit width | 0.007 | 0.173** | -0.039 | 0.226** | -0.227** | -0.220** | -0.164** | 0.219** | 0.065 |
| Index of fruit shape | -0.022 | -0.035 | 0.035 | -0.017 | 0.01 | 0.032 | 0.064 | -0.091 | -0.022 |
| Fruit weight | -0.110* | 0.165** | 0.002 | 0.240** | -0.229** | -0.254** | -0.217** | 0.253** | -0.021 |
| Nut vertical diameter | -0.175** | 0.027 | 0.114* | 0.135* | -0.155** | -0.168** | -0.137* | 0.08 | -0.061 |
| Nut transverse diameter | -0.071 | 0.098 | -0.056 | 0.106 | -0.149** | -0.083 | -0.064 | 0.081 | -0.055 |
| Nut lateral diameter | -0.04 | 0.096 | -0.048 | 0.118 | -0.195** | -0.141* | -0.107 | 0.125 | 0.022 |
| Mean diameter | -0.141* | 0.147* | -0.015 | 0.214** | -0.258** | -0.199** | -0.151* | 0.162* | -0.034 |
| Index of roundness | 0.112 | -0.026 | -0.096 | -0.108 | 0.063 | 0.083 | 0.075 | -0.052 | 0.049 |
| Shell thickness | -0.174** | -0.011 | 0.09 | 0.06 | -0.084 | -0.069 | -0.044 | -0.003 | -0.075 |
| Nut weight | -0.161* | 0.064 | 0.069 | 0.143* | -0.137* | -0.176** | -0.134* | 0.168** | -0.078 |
| Kernel weight | -0.194** | -0.021 | 0.169** | 0.106 | -0.123 | -0.082 | -0.014 | -0.106 | -0.165** |
| Kernel rate | 0.01 | 0.091 | -0.027 | 0.097 | -0.053 | -0.130* | -0.11 | 0.221** | -0.017 |
| Crude fat | -0.015 | 0.117 | 0.045 | 0.195** | -0.179** | -0.294** | -0.365** | 0.088 | -0.005 |
| Linoleic acid | 0.194* | -0.107 | -0.145 | -0.243** | 0.305** | 0.318** | 0.258** | -0.340** | 0.159 |
| Alpha-linolenic acid | -0.156 | -0.03 | 0.287** | 0.156 | -0.196* | -0.286** | -0.228* | 0.068 | -0.034 |
| Oleic acid | -0.049 | 0.09 | -0.081 | 0.066 | -0.086 | -0.039 | -0.034 | 0.208* | -0.101 |
| Amino acid | 0.061 | -0.039 | 0.025 | -0.038 | 0.005 | 0.021 | 0.093 | 0.052 | -0.082 |
| Vitamin_B6 | 0.413** | -0.096 | -0.13 | -0.217* | 0.304** | 0.460** | 0.507** | -0.404** | 0.192* |
| Vitamin E | -0.138 | -0.334** | 0.005 | -0.444** | 0.344** | 0.189* | 0.057 | -0.194* | -0.167 |
| Vitamin A | 0.105 | -0.034 | 0.145 | 0.054 | 0.002 | 0.041 | 0.153 | -0.105 | 0.123 |
| Fe | -0.178 | -0.082 | -0.084 | -0.179 | 0.159 | 0.142 | 0.103 | -0.174 | -0.395** |
| Zn | -0.328** | -0.188* | -0.076 | -0.313** | 0.238** | 0.13 | 0.003 | -0.125 | -0.446** |
| Ca | -0.077 | -0.102 | 0.182* | -0.018 | -0.146 | -0.343** | -0.397** | 0.003 | -0.113 |
| Mg | 0.213* | 0.200* | -0.114 | 0.185* | -0.320** | -0.314** | -0.393** | 0.084 | -0.028 |
| P | 0.102 | 0.119 | -0.016 | 0.141 | -0.313** | -0.396** | -0.486** | 0.063 | -0.1 |

Abbreviations for geographic/ecological conditions: ALT, altitude; AP, annual precipitation; AT5:accumulated temperature above 5℃; AT10:Accumulated temperature above 10℃; DL: day length; HLJ: Heilongjiang Province; JL: Jilin Province; LAT, latitude; LON, longitude; MAT, annual mean air temperature. *: p < 0.05; **: p < 0.01.
